# Preface to “Advances in Bio-Based and Biodegradable Polymeric Composites”

**DOI:** 10.3390/polym14142926

**Published:** 2022-07-20

**Authors:** Evgenia G. Korzhikova-Vlakh

**Affiliations:** Institute of Macromolecular Compounds, Russian Academy of Sciences, Bolshoy pr. 31, 199004 St. Petersburg, Russia; vlakh@mail.ru

The use of polymers for various purposes is increasing every year. In this regard, the production of polymeric materials from natural renewable sources, as well as the obtention of biodegradable polymeric materials and their recycling, comes to the fore in the packaging and disposable products industry. In addition, bio-based and biodegradable materials are becoming more and more prominent in medical practice as implants, regenerative and wound-healing materials, etc. Thus, modern environmental requirements, as well as the introduction of new technologies in biomedicine, pose the challenge to develop innovative “green” polymeric materials with the desired properties. The doping of polymeric materials with various fillers to prepare the composites makes it possible to adjust and improve their properties, such as mechanical and physicochemical characteristics, biological performance, degradation rate, conductivity, etc., in a targeted manner. The combination of bio-based and biodegradable polymers with fillers/reinforcements of various chemistries, dimensions, and geometries can lead to a higher level of diversity provided by composite materials as compared to just polymeric ones. 

Given the growing interest in polymeric composite materials, this Special Issue summarizes the latest advances in science and technology of bio-based and biodegradable composites, including the production, characterization, and application of such composites, as well as theoretical studies in the field. This Special Issue contains a number of original articles and reviews. The first group of papers reports on the use of poly (lactic acid), poly (ε-caprolactone), poly (butylene succinate), poly (vinyl alcohol), chitosan, pectin, lignin-epoxide, and corn starch as matrix polymers, and nanocellulose, bacterial cellulose, wood fibers, multiwalled carbon nanotubes, and silver, CaCO_3_ and ZnO nanoparticles as fillers. In turn, reviews are focused on overviewing the modification of cellulose and waste natural polymers as fillers, the production of conductive polymer composites for biomedical and biosensor technologies, and advances in the production of polymer composite packaging materials with antibacterial properties.

